# Predictors of disease complications and treatment outcome among patients with chronic suppurative otitis media attending a tertiary hospital, Mwanza Tanzania

**DOI:** 10.1186/s12901-015-0021-1

**Published:** 2016-01-07

**Authors:** Martha F. Mushi, Alfred E. Mwalutende, Japhet M. Gilyoma, Phillipo L. Chalya, Jeremiah Seni, Mariam M. Mirambo, Stephen E. Mshana

**Affiliations:** Department of Microbiology and Immunology, Weill Bugando School of Medicine, Catholic University of Health and Allied Sciences, P. O. BOX 1464, Mwanza, Tanzania; Department of Surgery, Weill Bugando School of Medicine, Catholic University of Health and Allied Sciences, Mwanza, Tanzania

## Abstract

**Background:**

Chronic suppurative otitis media (CSOM) is a major health problem in developing countries causing hearing loss and life threatening complications. Early and effective treatment based on the knowledge of causative micro-organisms and predictors of outcome are crucial in preventing these associated complications. This study was conducted to determine the predictors of CSOM complications, treatment outcome and antimicrobial susceptibility of pathogens, thus providing essential evidence to formulate a policy for management of CSOM.

**Methods:**

This was a prospective hospital based cross sectional study involving 301 patients attending Ear Nose and Throat (ENT) clinics at Bugando Medical Centre (BMC) between October 2013 and March 2014. A standardized data collection tool was used to collect demographics and clinical characteristics of patients with CSOM. Ear swabs were collected using sterile cotton swabs and transported to the laboratory for culture and antibiotic susceptibility testing.

**Results:**

Out of 301 patients with CSOM; 187 (62.1 %) had positive aerobic culture within 48 h of incubation. Disease complications and poor treatment outcome were observed in 114 (37.8 %, 95 % CI; 32.2–43.3) and 46 (15.3 %, 95 % CI; 11.2–19.3) respectively. On multivariate logistic regression analysis factors found independently to predict both disease complications and poor treatment outcome were otalgia, being infected by multi drug resistant bacteria and being HIV positive. Prolonged illness duration before seeking medical attention was also found to be associated with disease complications (OR 1.029, 95 % CI 1.007–1.05, *p* = 0.01). A total of 116 (61 %) of gram negative bacteria were isolated. Of 34 *Staphylococcus aureus*, 14 (41 %) were found to be methicillin resistant *Staphylococcus aureus* (MRSA) while of 116 g negative enteric bacteria, 49 (42 %) were extended spectrum beta lactamases producers (ESBL).

**Conclusions:**

Findings of this study suggest that positive HIV status, infection due to multidrug resistant pathogens and otalgia are significantly associated with disease complications and poor treatment outcome. Of great importance this study confirms that prolonged illness duration without seeking medical attention significantly predicts disease complications. Urgent preventive measures and laboratory guided early treatment are necessary to reduce complications associated with CSOM.

## Background

Chronic suppurative otitis media (CSOM) is defined as chronic inflammation of the middle ear in presence of the tympanic membrane perforation and discharge/otorrhoea for more than 6 weeks to 3 months [1, 2]. Chronic suppurative otitis media constitutes a major public health problem worldwide and it is associated with high morbidity [3]. Its incidence in developing countries is as high as 46 % and is common in children with low social economic status [4, 5]. Due to poor health seeking behavior and unavailability of ear, nose and throat (ENT) in primary health services, majority of these patients presents late to the tertiary hospitals [6, 7]. Chronic suppurative otitis media has been documented to be the commonest cause of preventable hearing loss [8–12]. *Pseudomonas aeruginosa*, *Staphylococcus aureus*, *Proteus mirabilis*, *Klebsiella pneumonia* and *Escherichia coli* have been found to be the commonest isolates causing CSOM in many studies [13, 14].

Despite the fact that CSOM constitutes a major cause of ENT clinic visits in Tanzania, little is known regarding disease complications, treatment outcome, spectrum of pathogens and their susceptibility pattern [15]. Lack of treatment guidelines and information regarding predictors of disease in most centres in developing countries like Tanzania make the management of CSOM more challenging. Hence, this study was performed in order to provide predictors of disease complications and poor treatment outcome, local data on aetiology and susceptibility pattern that will help in policy formulation and reduction of morbidity and mortality associated with CSOM.

## Methods

This was prospective hospital based cross sectional study conducted between October 2013 and March 2014 at ENT and surgical wards of the Bugando Medical Centre (BMC). The ENT clinic at BMC attends 632 patients annually. The protocol to conduct this study was approved by the Joint CUHAS/BMC Research, Ethics and publication committee.

All patients aged more than one year who presented with ear discharge for more than 6 weeks and tympanic membrane perforation were included into the study. All patients on topical ear antibiotics for 5 days or more at ENT clinic and surgical wards were excluded. All recruited patients were managed according to the routine treatment protocol of BMC and followed for 14 weeks.

The diagnosis of CSOM and its complications was confirmed by history taking to elicit the symptoms of ear problem like ear pain, ear discharge and duration of discharge [16]. Physical examination (otoscopic examination for verification of discharge and tympanic perforation as well as tuning fork examination –Rinne’s test and Weber’s test for assessment of hearing loss) were performed as previously described [1].

### Specimen collection and processing

Pus was aseptically collected using sterile cotton swabs (Heinz Herenz Hamburg, Germany) and transported to the laboratory using Stuart transport media (HiMedia Laboratories Pvt. Ltd, Mumbai, India) within an hour of collection.

The specimens were inoculated on Chocolate, blood and MacConkey agar plates (Oxoid, Basingstoke Hampshire RG24 8PW, UK). The culture plates were incubated (aerobically) at 37 °C for 24–48 hours before colonial morphologies were interpreted [17]. In house biochemical tests (catalase, coagulase, DNase, citrate, triple sugar iron agar, oxidase, urease, sulphur, indole and motility all from HiMedia, India) were performed to identify the isolates [17]. In case of ambiguous results, commercial available biochemical tests API 20E and API 20NE (Biomerieux, Marcy l’Etoile, France) were used for the confirmation. Susceptibility testing was performed following CLSI 2011 guidelines [18]. The following antibiotics were used: ciprofloxacin (CIP) 5 μg, trimethoprim**/**sulphamethoxazole (SXT) 1.25/23.75 μg, gentamicin (CN/GEN) 10 μg, ampicillin (AMP) 10 μg, vancomycin (VA) 30 μg, erythromycin (E) 15 μg, tetracycline 30 μg (Oxoid, UK) and cefoxitin (30 μg) to detect MRSA for gram positive bacteria while for gram negative bacteria ciprofloxacin (CIP) 5 μg, trimethoprim/sulphamethoxazole (SXT) 1.25/23.75 μg, gentamicin (CN/GEN) 10 μg, meropenem (MEM) 10 μg, amoxicillin/clavulanic acid (AMC) 20/10 μg, ceftriaxone (CRO) 30 μg and ceftazidime (CAZ) 30 μg (Oxoid, UK) were tested. Extended spectrum beta lactamases (ESBL) producing bacteria was detected as described previously [17].

### HIV testing

After provider initiated counselling and testing (PICT), HIV testing was performed using Tanzania National Rapid test algorithms protocol [19]. CD4 count was performed using FACS CALIBUR (BD Biosciences, USA), to all patients who were found to be HIV positive to determine severity of immune suppression.

### Patient’s management

On admission all patients were screened for diseases complications (Unfavorable evolution of a disease) such as hearing loss, mastoditis and intracranial infections. All patients suspected with mastoid bone osteomyelitis were investigated using plain film radiography of mastoid region. For these patients with chronic mastoiditis surgical management was added on top of conservative management which was done to the rest of patients. The conservative management involved ear wicking and use of topical antimicrobial agents based on BMC treatment guidelines which advocate the ciprofloxacin ear drops and boric acid drops while surgical management included surgical debridement/mastoidectomy and incision & drainage. All patients were followed on clinic visits of once per month for 14 weeks to determine treatment outcome as evidenced by persistence of ear discharge. In some cases phone calls were done to ascertain the progress after 14 weeks. In the second visit ear drops were changed based on culture and sensitivity results.

### Statistical data analysis

Data analysis was done using STATA version 11. Data were summarized in form of proportions, frequent tables and bar graph for categorical variables. Means (standard deviation) and median (inter quartile range) were used to summarize continuous variables. Univariate followed by multivariate logistic regression analysis were done to determine predictors of treatment outcome. Predictors investigated included; illness duration, age, sex, type of isolates, susceptibility pattern, smoking, HIV status, smell of discharge, otalgia and ear involved. Odds ratios with respective 95 % confidence interval (CI) were reported. Predictors with a p-value of less than 0.05 were considered statistically significant.

## Results

### Patients characteristics

A total of 301 patients with CSOM were studied, their mean age was 33.7 ± 17.9 years. Of the studied population 165 (54.8 %) were females (Table 1). Majority of patients 115 (38.2 %) in this study were small scale farmers, followed by students 65 (21.6 %) and the rest were employed (Table 1). Out of 301 patients, 155 (51.5 %) came from rural areas and 146 (48.5 %) from urban areas. Among the study participants; 24 (8.0 %) patients presented with history of pre morbid illness, of which diabetes were reported in 22/24 (92 %) and cancer in 2 (0.7 %). Out of 301 participants, 13 (4.3 %) were found to be HIV positive (Table 2).

**Table 1:** Demographic characteristics of patients

Patients’ characteristic	Number of patients	(%)
Sex		
Female	165	54.8
Male	136	45.2
Age (years)		
1–10	31	10.30
11–20	52	17.28
21–30	51	16.94
31–40	59	19.60
41–50	55	18.27
>50	33	17.61
Occupation		
Business	53	17.61
Students	65	21.59
Peasants	115	38.21
Public servants	49	16.28
NA/children	19	6.31
Education level		
Pre primary	23	7.64
Primary	128	42.52
Secondary	110	36.54
Tertiary	37	12.29
No formal education	3	1.00
Residency		
Rural	155	51.5
Urban	146	48.5
Smoking		
Yes	41	13.62
No	260	86.38
HIV status		
Positive	13	4.32
Negative	277	92.03
Refused to test	11	3.65
Co-morbidity		
Yes	24	8.0
No	277	92.0

**Table 2:** Distribution of clinical presentation in the study population

Patients’ characteristic	Number of patients	(%)
Ear affected		
Right	161	53.49
Left	122	40.53
Bilateral	18	5.98
Duration of illness/months		
0–6	66	21.93
7–12	57	18.94
13–24	123	40.86
25–36	44	14.62
>36	11	3.65
Presenting symptoms		
Smell	34	11.33
Otalgia	134	44.82
Itching	74	24.58
Tinnitus	97	32.23
^a^Others	5	1.66

^a^Vertigo 3, irritability 1, Nasal discharge 1

At the time of enrolment, 114 (37.9 %) of patients had hearing loss (99 conductive, 4 sensory neural and 11 mixed) and 5 (1.7 %) had mastoiditis. All patients with mastoiditis also had conductive hearing loss.

All patients were treated conservatively, 16 (5.3 %) surgical debridement was added and 5 (1.7 %) also underwent mastoidectomy.

### Isolates and susceptibility pattern

Out of 301 patients with CSOM, 187 (62.1 %) had positive aerobic culture within 48 h of incubation. Of these gram negative bacteria 116 (61 %) formed majority of isolates predominated by *Pseudomonas* spp. 56 (29.5 %) Table 3. Of 36 gram positive bacteria; 34 (94 %) were identified as *Staphylococcus aureus* with 14 (41 %) identified as methicillin resistant *Staphylococcus aureus* (MRSA). *Pseudomonas* spp. were 52, 47, 9, 2 and 0 % resistant to amoxicillin/clavulanic acid, ceftazidime, gentamicin, meropenem and ciprofloxacin respectively Table 3. Of 116 g negative enteric bacteria, 49 (42 %) were found to be ESBL.

**Table 3:** Resistance profile of bacterial isolates in the study population

Bacterial isolate	E	VA	SXT	TE	CIP	GEN	CAZ	AMC	CRO	MEM	AMP
Pseudomonas spp. (56)	-	-			0 %	9 %	47 %	95 %		2 %	100 %
S. aureus (34)	31 %	3 %	74 %	6 %	4 %	11 %	-	-	-	-	
Proteus spp. (27)	-	-	-	-	0 %	4 %	8 %	70 %	7 %	4 %	100 %
E. coli (22)	-	-	-	67 %	10 %	53 %	70 %	90 %	70 %	0 %	91 %
* Other gram negative (13)	-	-	75 %	32	33 %	63 %	67 %	50 %	75 %	25 %	100 %

E, VA, OX were tested for gram positive bacteria and GEN, CIP, AMC were tested for both gram positive and gram negative bacteria and CAZ, MEM, CRO were tested for gram negative bacteria

Abbreviation: AMC, AMP CAZ, CRO, E, GEN, CIP, SXT, TE, OX, VA and MEM stand for amoxicillin/clavulanic acid, ampicillin, ceftazidime, ceftriaxone, erythromycin, gentamicin, ciprofloxacin, trimethoprim/sulphamethoxazole, tetracycline, oxacillin, vancomycin and meropenem respectively

*Other gram negative: *Klebsiella* spp. (8), *Acaligenesis* spp. (4) and *Acinetobacter* spp. (1)

### Factors predicting disease complications

Disease complications were observed in 114/301 (37.8 %, 95 % CI; 32.2–43.3) patients. Conductive hearing loss was the commonest 99 (86.8 %) complication observed. Of 288 patients with negative HIV sero-status, 105 (36.4 %) had disease complications compared to 9 (69.2 %) of 13 patients with positive HIV sero-status (*p* = 0.028). It was also observed that as illness duration increases by one month the risk of getting disease complications increases by 3 %. On multivariate logistic regression analysis increase in illness duration (OR 1.03, 95 % CI; 1.007–1.05; *p* = 0.01), being infected by multi drug resistant bacteria (OR 1.86, 95 % CI; 1.04–3.3; *p* = 0.035), being HIV positive (OR 4.3, 95 % CI; 1.17–15.6; *p* = 0.028) and otalgia (OR 1.9, 95 % CI; 1.14–3.18; *p* = 0.013) were independently factors found to predict disease complications Table 4.

**Table 4:** Factors predicting complications of CSOM

Variable	Univariate			Multivariate	
	Complication n (%)	OR (95 % CI)	P value	OR (95 % CI)	P value
Age	35 ± 17.82	1.006 (0.99–1.011)	0.318	1.006 (0.997–1.02)	0.443
Sex					
Female (165)	66 (40)	1			
Male (136)	48 (35.3)	0.81 (0.511–1.3)	0.402	0.88 (0.49–1.55)	0.679
Occupation					
No (199)	31 (15.6)	1			
Yes (102)	15 (14.7)	0.93 (0.47–1.8)	0.842	0.8 (0.37–1.7)	0.568
Ill duration	18 IQR (9–27)	1.029 (1.008–1.05)	0.005	1.03 (1.007–1.05)	0.010
Isolates					
NBG (175)	67 (38.29)	1			
GPB (35)	21 (60)	2.41 (1.15–5.07)	0.02		
GNB (91)	45 (49.45)	1.57 (0.94–2.63)	0.081		
MDR					
No (229)	81 (35.6)	1			
Yes (72)	33 (45.8)	1.54 (0.90–2.6)	0.112	1.86 (1.044–3.3)	0.035
HIV					
No (288)	105 (36.4)	1			
Yes (13)	9 (69.2)	3.92 (1.17–13.)	0.026	4.3 (1.17–15.6)	0.028
Smoking					
No (260)	100 (38.5)	1			
Yes (41)	14 (34.2)	0.83 (0.415–1.6)	0.597	0.80 (0.339–1.88)	0.611
Smell					
No (267)	98 (36.7)	1			
Yes (34)	16 (47.1)	1.53 (0.747–3.4)	0.244	1.62 (0.73–3.56)	0.234
Otalgia					
No (167)	52 (31.1)	1			
Yes (134)	62 (46.30	1.98 (1.2–3.05)	0.007	1.9 (1.14–3.18)	0.013
Itching					
No (227)	90 (39.6)	1			
Yes (74)	24 (32.4)	0.7 (0.412–1.27)	0.267	0.66 (0.359–1.24)	0.205
Ear					
Left (122)	41 (33.6)	1			
Both (18)	13 (72.2)	5.13 (1.7–15.4)	0.003		
Right (161)	60 (37.2)	1.17 (0.72–1.92)	0.525	1.07 (0.82–1.40)	0.573

NBG is no bacterial growth, GNB is gram negative bacteria, GPB is gram positive bacteria and MDR is multi drug resistance bacteria

Isolates were not analysed on multivariate logistic regression analysis because it has collinearity with other factor like MDR

### Factors predicting poor treatment outcome

In the current study poor treatment outcome defined by persistent otorrhoea was observed in 46 (15.3 %, 95 % CI; 11.2–19.3) of patients. Of HIV negative patients 38 (13.2 %) had poor treatment outcome compare to 8 (61.5 %) of HIV positive patients *p* < 0.001. Out of 134 patients with otalgia, 28 (20.9 %) had poor treatment outcome compared to 18/167 (10.8 %) of those without otalgia (*p* = 0.01) On multivariate logistic regression analysis being infected by multi drug resistant bacteria (OR 3.6, 95 % CI; 1.7–7.59; *p* = 0.001), being HIV positive (OR 11.8, 95 % CI; 3.24–43.1; *p* < 0.001) and otalgia (OR 3.3, 95 % CI; 1.56–7.02; *p* = 0.002) were independently factors found to predict poor treatment outcome Table 5.

**Table 5:** Factors predicting poor treatment outcome of CSOM

Variable	Univariate			Multivariate	
	Otorrhoea: n (%)	OR (95 % CI)	P value	OR (95 % CI)	P value
Age	34.42 ± 18.04	1.004 (0.98–1.019)	0.877	1.001 (0.97–1.02)	0.956
Sex					
Female (165)	21 (12.7)	1			
Male (136)	25 (18.4)	1.5 (0.82–2.9)	0.177	1.47 (0.66–3.2)	0.399
Occupation					
No (199)	31 (15.6)	1			
Yes (102)	15 (14.7)	0.93 (0.47–1.8)	0.842	0.8 (0.37–1.7)	0.568
Ill duration	18 IQR (9–24)	1.0087 (0.98–1.04)	0.507	0.99 (0.96–1.027)	0.797
Isolates					
NBG (175)	67 (38.29)	1			
GPB (35)	21 (60)	2.41 (1.15–5.07)	0.02		
GNB (91)	45 (49.45)	1.57 (0.94–2.63)	0.081		
MDR					
No (229)	27 (11.8)	1			
Yes (72)	19 (26.4)	2.68 (1.38–5.1)	0.003	3.6 (1.7–7.59)	0.001
HIV					
No (288)	38 (13.2)	1			
Yes (13)	8 (61.5)	10.5 (3.2–33.8)	0.000	11.8 (3.24–43.1)	0.000
Smoking					
No (260)	37 (14.2)	1			
Yes (41)	9 (21.9)	1.7 (0.74–3.8)	0.206	1.08 (0.37–3.14)	0.883
Smell					
No (267)	38 (14.2)	1			
Yes (34)	6 (23.5)	1.85 (0.78–4.39)	0.27	2 (0.74–5.69)	0.164
Otalgia					
No (167)	18 (10.8)	1			
Yes (134)	28 (20.9)	2.18 (1.49–3.8)	0.017	3.3 (1.56–7.02)	0.002
Itching					
No (227)	30 (13.2)	1			
Yes (74)	16 (21.6)	1.8 (0.92–3.55)	0.084	1.82 (0.81–4.10)	0.144
Ear					
Left (122)	18 (14.7)	1			
Both (18)	1 (5.56)	0.3 (0.04–2.7)	0.309		
Right (161)	27 (16.77)	1.1 (0.60–2.2)	0.646	1.08 (0.74–1.58)	0.658

NBG is no bacterial growth, GNB is gram negative bacteria, GPB is gram positive bacteria and MDR is multi drug resistance bacteria. Isolates were not analysed on multivariate logistic regression analysis because it has collinearity with other factor like MDR

## Discussion

### Demographic and clinical presentations

In the current study, though not statistically significant CSOM was a common problem in third and fourth decades of life. Similar results were reported in University hospitals in Singapore by Loy et al. [20, 21] in Nepal. This could be explained by a possibility of persistence silent disease and failure to present early to hospital with ENT services as previously reported [22–24] and confirmed in this study.

### Predictors of disease complication and poor treatment outcome

HIV seropositive among patients with CSOM has been reported to be associated with severe disease and poor treatment outcome [23, 25]. This has been confirmed in the current study whereby HIV positive patients were 4.3 and 11.8 times more to have disease complications and poor treatment outcome than HIV negative patients. Though not statistically significant in this study, patients who were smoking had 8 % more chance to have poor treatment outcome than non-smokers. The influence of smoking has been documented previously [8, 22] and this could be due to the fact that smoking cause irritations to nasal passage causing thickening of nasal mucosa with mucous which favours bacteria growth. Also smoking has been proven to weaken immune system hence recurrent upper respiratory tract infections including otitis media [26].

This study confirms what has been documented previously regarding the contribution of prolonged illness duration in bringing disease complication such as conductive hearing loss [8, 27]. Prolonged illness duration can be due to ignorance, treatment at home, cost, poverty and mainly poor infrastructures as long as ENT services are concerned in many developing countries such as Tanzania.

Other factors found independently to predict disease complications and poor treatment outcome were otalgia and infection due to multi drug resistant bacteria. The presence of otalgia could explain severe inflammation associated with extensive pathology leading to persistence of the pathology even after treatment. More extensive management and prolonged treatment might be necessary in patients with otalgia. Invasive infections with multi drug resistant pathogens have been found to be associated with increased morbidity and mortality [28]. In the present study patients infected with multi drug resistant pathogens were 1.86 and 3.6 times more likely to have disease complications and poor treatment outcome than those infected with sensitive bacteria. These findings underscore the importance of empirical treatment derived from local susceptibility data.

### Pathogens and susceptibility patterns

In the present study bacterial growth rate was lower compared to previously studies done in Ethiopia, Philippines, Nigeria, Kenya and India [3, 11, 12, 24, 29] respectively. The relatively low culture positive rate in this study could be due to prior use of antibiotics and inability to perform anaerobic culture.

As documented previously [15, 29, 30] in the present study gram negative bacteria formed majority of isolates, predominated by *Pseudomonas* spp. Natural habitat, minimum nutritional requirement and ability to colonize moist areas of the body support our findings [31].

*Pseudomonas* spp. isolated in our study were all susceptible to ciprofloxacin and 91 % sensitive to gentamicin; these results still give assurance on the usefulness of these agents as effective first line topical antibiotics in treatment for CSOM. In contrast to *Pseudomonas* spp. isolated from wounds in the same hospital these isolates are generally more sensitive. This could be due to the fact that patients from this study were coming from the community and those in previous study were from hospital [32]. Hospital isolates have been found to be more resistant than community isolates [18]. Methicillin Resistant *Staphylococcus aureus* (MRSA) was detected in 41 % which concurs with studies undertaken previously in Nigeria [33, 34]. As limitation, some pathogens observed in this study might not be the true cause of the pathology because of the chronic nature of the process leading to the pathology.

## Conclusions

CSOM due to multi drug resistant bacteria is common in our setting. Majority of patients with prolonged illness duration, otalgia, infected with multi drug resistant bacteria and those with positive HIV status poorly respond to treatment and tend to present with disease complications. Guidelines for management of CSOM in developing countries are needed so that associated complications can be reduced.

## References

[1] Acuin J (2004). Chronic suppurative otitis media: burden of illness and management options.

[2] Kenna MA (1994). Treatment of chronic suppurative otitis media. Otolaryngol Clin North Am.

[3] Abera B, Kibret M (2011). Bacteriology and antimicrobial susceptibility of otitis media at dessie regional health research laboratory, Ethiopia. Ethiop J Health Dev.

[4] Ercan P, Öncel S, Karagöz U, Sener G, Tatar B (2008). Demonstration of bacterial biofilms in chronic otitis media. Mediterranean J Otol.

[5] Verma AK, Vohra A, Maitra A, Banerjee M, Singh R, Mittal SK (1995). Epidemiology of chronic suppurative otitis media and deafness in a rural area and developing an intervention strategy. Indian J Pediatr.

[6] Minja BM, Moshi NH, Ingvarsson L, Bastos I, Grenner J (2006). Chronic suppurative otitis media in Tanzanian school children and its effects on hearing. East Afr Med J.

[7] Vikram B, Khaja N, Udayashankar S, Venkatesha B, Manjunath D (2008). Clinico-epidemiological study of complicated and uncomplicated chronic suppurative otitis media. J Laryngol Otol.

[8] WHO. Child and adolescent health and development, prevention of blindness and deafness. Chronic suppurative otitis media Burden of Illness and management options. 2004, ISBN 92-4-159158 7.

[9] Alabbasi AM, Alsaimary IE, Najim JM (2010). Prevalence and patterns of chronic suppurative otitis media and hearing impairment in Basrah city. J Med Med Sci.

[10] Salisu AD (2010). Otology practice in a Nigerian tertiary health institution: a 10-year review. Ann Afr Med.

[11] Ayson NP, Lopez GJ, Llanes EG (2006). Chronic suppurative otitis media: bacteriology and drug sensitivity patterns at the Quirino Memorial Medical Center: a preliminary study. Philippine J Otolaryngol Head Neck Surg.

[12] Bakari AA, Adoga AA, Afolabi OA, Kodiya AM, Ahmad BM (2010). Pattern of chronic suppurative otitis media at the National Ear Care Centre Kaduna, Nigeria. J Med Trop.

[13] Pelikan Z (2006). Chronic Otitis Media (Secretory) and nasal allergy. Scr Med (BRNO).

[14] Singh AH, Basu R, Venkatesh A (2012). Aerobic bacteriology of chronic suppurative otitis media in Rajahmundry, India. Biol Med.

[15] Moshi NH, Minja BM, Ole-Lengine L, Mwakagile DS (2000). Bacteriology of chronic otitis media in Dar es Salaam, Tanzania. East Afr Med J.

[16] ALHO OP (1990). The validity of questionnaire reports of a history of acute otitis media. Am J Epidemiol.

[17] Mshana SE, Kamugisha E, Mirambo M, Lyamuya E (2009). Prevalence of multi resistant gram negative organism in a tertiary hospital in Mwanza Tanzania. BMC Res Notes.

[18] Wayne PA. Clinical and Laboratory Standards Institute(CLSI): Performance standards for antimicrobial disk susceptibility tests.19th edition Approved standard. CLSI Document 2009, M100-S19:29.

[19] National AIDS control Programme (NACP). National guidelines for management of HIV/AIDS. 4th ed. Tanzania. 2012.

[20] Loy A, Tan A, Lu P (2002). Microbiology of chronic suppurative otitis media in Singapore. Singapore Med J.

[21] Shrestha B, Amatya R, Shrestha I, Ghosh I (2012). Microbiological profile of chronic supurative otitis media. Nepalese J Ent Head Neck Surg.

[22] Monasta L, Ronfani L, Marchetti F, Montico M, Vecchi Brumatti L, Bavcar A (2012). Burden of disease caused by otitis media: systematic review and global estimates. PLoS One.

[23] Taipale A, Pelkonen T, Taipale M, Bernardino L, Peltola H, Pitkäranta A (2011). Chronic suppurative otitis media in children of Luanda, Angola. Acta Paediatr.

[24] de Aquino JEAP, Pererira SH, de Aquino JNP, Neto RG, Carvalho MR, Cruz Filho NA (2009). Bacterial findings found in the chronic otitis media secretion: comparative study between cholesteatoma (OMCC) and Simple Chronic Otitis Media (SCOM).

[25] Karpakis J, Rabie H, Howard J, van Rensburg AJ, Cotton M (2008). Otorrhoea is a marker for symptomatic disease in HIVinfected children. S Afr Med J.

[26] Landrigan PJ, Carlson JE, Bearer CF, Cranmer JS, Bullard RD, Etzel RA (1998). Children’s health and the environment: a new agenda for prevention research. Environ Health Perspect.

[27] Smith AW (2001). WHO activities for prevention of deafness and hearing impairment in children. Scand Audiol.

[28] Kayange N, Kamugisha E, Mwizamholya DL, Jeremiah S, Mshana SE (2010). Predictors of positive blood culture and deaths among neonates with suspected neonatal sepsis in a tertiary hospital, Mwanza-Tanzania. BMC Pediatr.

[29] Prakash R, Juyal D, Negi V, Pal S, Adekhandi S, Sharma M (2013). Microbiology of chronic suppurative otitis media in a tertiary care setup of Uttarakhand state, India. N Am J Med Sci.

[30] Iqbal K, Khan MI, Satti L (2011). Microbiology of chronic otitis media: experience at Dera Ismail Khan. Gomal J Med Sci.

[31] Kong HH, Segre JA (2012). Skin microbiome: looking back to move forward. J Investig Dermatol.

[32] Moremi N, Mushi MF, Fidelis M, Chalya P, Mirambo M, Mshana SE (2014). Predominance of multi-resistant gram-negative bacteria colonizing chronic lower limb ulcers (CLLUs) at Bugando Medical Center. BMC Res Notes.

[33] Adoga A, Nimkur T, Silas O (2010). Chronic suppurative otitis media: socio-economic implications in a tertiary hospital in Northern Nigeria. Pan Afr Med J.

[34] Afolabi O, Salaudeen A, Ologe F, Nwabuisi C, Nwawolo C (2013). Pattern of bacterial isolates in the middle ear discharge of patients with chronic suppurative otitis media in a tertiary hospital in North central Nigeria. Afr Health Sci.

